# The maternal blood lipidome is indicative of the pathogenesis of severe preeclampsia

**DOI:** 10.1016/j.jlr.2021.100118

**Published:** 2021-09-20

**Authors:** Bing He, Yu Liu, Mano R. Maurya, Paula Benny, Cameron Lassiter, Hui Li, Shankar Subramaniam, Lana X. Garmire

**Affiliations:** 1 Department of Computational Medicine and Bioinformatics, University of Michigan, Ann Arbor, MI, USA; 2 Department of Bioengineering and San Diego Supercomputer Center, University of California San Diego, La Jolla, CA, USA; 3 University of Hawaii Cancer Center, Department of Epidemiology, Honolulu, HI, USA; 4 Departments of Computer Science & Engineering and Cellular & Molecular Medicine, University of California San Diego, La Jolla, CA, USA

**Keywords:** preeclampsia, lipidomics, metabolomics, pathway, biomarker, hypertension, maternal blood, pregnancy, machine learning, classification, ACN, acetonitrile, CE, cholesteryl ester, Cer, ceramide, KNN, K-nearest neighbor, LC3, light chain 3, LPC, lysophosphatidylcholine, LPE, lysophosphatidylethanolamine, OxPC, oxidized PC, OxPE, oxidized phosphatidylethanolamine, OxPL, oxidized phospholipid, PC, phosphatidylcholine, PE, phosphatidylethanolamine, PL, phospholipid, QC, quality control, RF, random forest, SOV, source of variation, TAG, triacylglycerol, WGCNA, weighted gene coexpression network analysis

## Abstract

Preeclampsia is a pregnancy-specific syndrome characterized by hypertension and proteinuria after 20 weeks of gestation. However, it is not well understood what lipids are involved in the development of this condition, and even less is known how these lipids mediate its formation. To reveal the relationship between lipids and preeclampsia, we conducted lipidomic profiling of maternal sera of 44 severe preeclamptic and 20 healthy pregnant women from a multiethnic cohort in Hawaii. Correlation network analysis showed that oxidized phospholipids have increased intercorrelations and connections in preeclampsia, whereas other lipids, including triacylglycerols, have reduced network correlations and connections. A total of 10 lipid species demonstrate significant changes uniquely associated with preeclampsia but not any other clinical confounders. These species are from the lipid classes of lysophosphatidylcholines, phosphatidylcholines (PCs), cholesteryl esters, phosphatidylethanolamines, lysophosphatidylethanolamines, and ceramides. A random forest classifier built on these lipids shows highly accurate and specific prediction (F1 statistic = 0.94; balanced accuracy = 0.88) of severe preeclampsia, demonstrating their potential as biomarkers for this condition. These lipid species are enriched in dysregulated biological pathways, including insulin signaling, immune response, and phospholipid metabolism. Moreover, causality inference shows that various PCs and lysophosphatidylcholines mediate severe preeclampsia through PC 35:1e. Our results suggest that the lipidome may play a role in the pathogenesis and serve as biomarkers of severe preeclampsia.

Preeclampsia is a pregnancy-specific syndrome that aspects 2–8% of pregnancies and is diagnosed when a pregnant woman presents with increased blood pressure and proteinuria (1). It is a leading cause of maternal, fetal, and neonatal mortality, especially in low-income and middle-income countries (2). Depending on the onset time, preeclampsia can be categorized as early onset preeclampsia (<34 weeks) and late onset preeclampsia. Early onset preeclampsia is a more severe form and associated with shallow placental implantation into the uterine wall and subsequent placental dysfunction (3). Alternatively, preeclampsia can be classified as mild or severe type based on the severity of the symptoms. For severe preeclampsia, the mothers often suffer from potentially fatal pathological manifestations, including hypertension, proteinuria, liver rupture, pulmonary edema, and kidney failure (4). Moreover, women who had preeclampsia previously have shown 2–3-folds' higher risks at developing cardiovascular diseases later in life (5). The adverse impacts on the fetus include intrauterine growth restriction and preterm delivery. Despite the severity of this condition, few effective treatments are available except expectant management. Delivery of the placenta is the only cure, but this is often coupled with preterm delivery of the fetus (6), who are more likely to have lifelong health issues, such as neurodevelopmental disorders and adult onset disorders (7).

Many efforts have been made to systematically understand the biological processes altered in this syndrome as well as identify potential biomarkers using genomics platforms, such as DNA methylation, transcriptomics, proteomics, and metabolomics (8, 9, 10). However, the systematic changes in lipids, which are more stable compared with other metabolites, are less studied for preeclampsia (11, 12, 13). Lipids have shown biomarker potential for many diseases (14, 15). Moreover, lipids can reflect the physiological or pathological status of a metabolic disease such as preeclampsia, as they are structural components of cell membranes, signaling mediators, and energy depots. Recent developments in mass spectrometric methods for lipidomics allow for untargeted measurements of hundreds of lipids simultaneously (16). For preeclampsia metabolomics research, one study found 11 lipid classes in the maternal blood of women with early onset preeclampsia different from those of healthy pregnant women (17). Another study identified a panel of 23 serum lipidomic biomarkers from 10 lipid classes to predict the risk of preeclampsia in a pregnancy cohort at 12–14 weeks of gestation (18). A different study found that the first trimester maternal plasma ceramide species (Cer 14:0) and SM species (SM 16:0 and SM 18:1) may be early biomarkers of preeclampsia occurrence (19). However, none of these studies systematically analyzed the potential molecular mechanisms underlying the identified lipids beyond biomarker modeling. Moreover, lipids that are linked to the occurrence and pathogenesis of severe preeclampsia have not been investigated yet.

Here, we conducted untargeted lipidomics profiling of maternal blood in a multiethnic cohort of severe preeclampsia (N = 44) patients and those with full-term healthy deliveries (N = 20). By combining LC-MS technology and advanced bioinformatics analysis, we provide novel insights into lipids and their pathways involved in preeclampsia, in addition to identifying new biomarkers for severe preeclampsia. Our analysis shows that a variety of lipids are altered in severe preeclampsia, and some are directly involved in causal mechanisms. These molecular changes coherently lead to dysregulated biological functions, such as insulin signaling and inflammation/infections. Oxidized phospholipids (OxPLs) are significantly coordinated and upregulated, presumably because of oxidative stress from hypoxia.

## Materials and methods

### Specimens

We obtained samples from RMATRIX Hawaii Biorepository, which obtained its own institutional review boards' approval. All the subjects gave informed consent. This study abides by the Declaration of Helsinki principles. About 44 maternal plasma samples from clinically diagnosed severe preeclampsia patients and 20 control samples (full-term deliveries) were selected. The clinical summary of the samples is provided in Table 1.

**Table 1:** Demographic and clinical characteristics in case and control groups

Characteristics	Control (n = 20)	Case (n = 44)	*P*^a^
	Mean (SD)s		
Maternal age, years	27.50 (6.52)	29.27 (6.79)	0.33
Prepregnancy BMI, kg/m^2^	29.37 (6.82)	29.28 (7.06)	0.96
Gestational age, weeks	39.10 (0.85)	35.82 (2.89)	4.80e-9
Parity			0.017
0	10	20	
1	1	13	
2	8	5	
3 and above	1	6	
Smoker			0.088
Yes	0	7	
No	20	37	
Maternal ethnicity			0.22
Asian	13	22	
Caucasian	2	6	
Latin	2	1	
Pacific Island	3	15	
Baby gender			0.42
Male	8	24	
Female	12	20	
Gestational diabetes			0.013
Yes	0	11	
No	20	33	
Chronic hypertension			0.085
Yes	1	11	
No	19	33	
Membrane rupture			0.25
Yes	9	12	
No	11	32	
Abruption			0.55
Yes	0	3	
No	20	41	
Neonatal malformations			0.55
Yes	0	3	
No	20	41	

a Categorical variables were compared using Fisher's exact test, whereas continuous variables were compared using *t* test.

### Reagents and internal standards

HPLC-grade acetonitrile (ACN) and dichloromethane were purchased from Sigma-Aldrich (St. Louis, MO), isopropanol (Optima—LC/MS grade) was purchased from Fisher (New Jersey, NJ), and methanol (LC-MS grade) was purchased from J.T. Baker. Water was obtained from a Millipore high-purity water dispenser (Billerica, MA). The following MS-grade lipid standards were obtained from Sigma-Aldrich: 1-heptadecanoyl-2-hydroxy-*sn*-glycero-3-phosphocholine lysophosphatidylcholine (LPC) (17:0/0:0), 1,2-diheptadecanoyl-*sn*-glycero-3-phosphocholine phosphatidylcholine (PC) (17:0/17:0), 1,2-diheptadecanoyl-*sn*-glycero-3-phosphoethanolamine phosphatidylethanolamine (PE) (17:0/17:0), 1,2-diheptadecanoyl-*sn*-glycero-3-phospho-l-serine (sodium salt) phosphatidylserine (17:0/17:0), *N*-heptadecanoyl-d-*erythro*-sphingosylphosphorylcholine 17:0 SM (d18:1/17:0), cholest-5-en-3ß-yl heptadecanoate 17:0 cholesteryl ester (CE), 1-palmitoyl-2-oleoyl-*sn*-glycerol 16:0-18:1 diglyceride/diacylglycerol, 1-heptadecanoyl-rac-glycerol 17:0 monoglyceride/monoacylglycerol, 1,2,3-triheptadecanoyl-glycerol triheptadecanoate 17:0 triacylglycerol (TAG), *N*-heptadecanoyl-d-*erythro*-sphingosine C17 Cer (d18:1/17:0), 1,2-diheptadecanoyl-*sn*-glycero-3-phosphate (sodium salt) 17:0 phosphatidic acid, 1,2-diheptadecanoyl-*sn*-glycero-3-phospho-(1′-*rac*-glycerol) (sodium salt) 17:0 phosphatidylglycerol, 1-heptadecanoyl-2-(5Z,8Z,11Z,14Z-eicosatetraenoyl)-*sn*-glycero-3-phospho-(1′-myo-inositol) (ammonium salt) 17:0-20:4 phosphatidylinositol, 1,3(d5)-dinonadecanoyl-2-hydroxy-glycerol diglyceride/diacylglycerol d^5^-(19:0/0:0/19:0), and glyceryl tri(palmitate-d^31^) triglyceride d^31^.

### Sample preparation

The lipids were extracted from plasma using a modified Bligh-Dyer method (20) using a 2:2:2 ratio volume of methanol:water:dichloromethane at room temperature after spiking internal standards (supplemental Table S1) The organic layer was collected and completely dried under nitrogen. Before MS analysis, the dried lipid extract was reconstituted in 100 μl of buffer B (10:85:5 ACN/isopropyl alcohol/water) containing 10 mM ammonium acetate and subjected to LC/MS.

### Internal standards and quality controls

Quality control (QC) samples were prepared by pooling equal volumes of each sample and injected at the beginning and the end of each analysis and after every 10 sample injections to provide a measurement of the system's stability and performance as well as reproducibility of the sample preparation method (21).

Two kinds of controls were used to monitor the sample preparation and MS. To monitor instrument performance, 10 μl of a dried matrix-free mixture of the internal standards reconstituted in 100 μl of buffer B (85% isopropyl alcohol:10% ACN:5% water in 10 mM NH_4_OAc) was analyzed. As additional controls to monitor the profiling process, an equimolar mixture of 15 authentic internal standards and a characterized pool of human plasma and test pool (a small aliquot from the all preeclampsia plasma used in this study) (extracted in tandem with preeclampsia plasma) were analyzed along with the preeclampsia plasma samples. Each of these controls was included several times into the randomization scheme such that sample preparation and analytical variability could be monitored constantly.

### Data-dependent LC-MS/MS for measurements of lipids

Chromatographic separation was performed on a Shimadzu CTO-20A Nexera X2 UHPLC systems equipped with a degasser, binary pump, thermostatted autosampler, and column oven (all components manufactured by Shimadzu [Canby, OR]). The column heater temperature was maintained at 55°C, and an injection volume of 5 μl was used for all analyses. For lipid separation, the lipid extract was injected onto a 1.8-μm particle diameter, 50 × 2.1 mm id Waters Acquity HSS T3 column (Waters, Milford, MA). Elution was performed using ACN/water (40:60, v/v) with 10 mM ammonium acetate as solvent A and ACN/water/isopropanol (10:5:85 v/v) with 10 mM ammonium acetate as solvent B. For chromatographic elution, we used a linear gradient beginning with 60% solvent A and 40% solvent B. The gradient was ramped in a linear fashion to 98% solvent B over the first 10 min and was held at 98% solvent B for 7 min. Thereafter, the composition was returned to 40% solvent B and 60% solvent A and held for 3 min. The flow rate used for these experiments was 0.4 ml/min, and the injection volume was 5 μl. The column was equilibrated for 3 min before the next injection and ran at a flow rate of 0.400 μl/min for a total run time of 20 min.

MS data acquisition for each sample was performed in both positive and negative ionization modes using a TripleTOF 5600 equipped with a DuoSpray ion source (AB Sciex, Concord, Canada). Column effluent was directed to the ESI source, and voltage was set to 5,500 V for positive ionization and 4,500 V for negative ionization mode. The declustering potential was 60 V, and source temperature was 450°C for both modes. The curtain gas flow, nebulizer, and heater gas were set to 30, 40, and 45, respectively (arbitrary units). The instrument was set to perform one TOF MS survey scan (150 ms) and 15 MS/MS scans with a total duty cycle time of 2.4 s. The mass range of both modes was *m/z* 50–1,200. Acquisition of MS/MS spectra was controlled by the data-dependent acquisition function of the Analyst TF software (AB Sciex, Concord, Canada) with application of following parameters: dynamic background subtraction, charge monitoring to exclude multiply charged ions and isotopes, and dynamic exclusion of former target ions for 9 s. Collision energy spread of 20 V was set, whereby the software calculated the CE value to be applied as a function of *m/z*.

A DuoSpray source coupled with an automated calibration system (AB Sciex, Concord, Canada) was utilized to maintain mass accuracy during data acquisition. Calibrations were performed at the initiation of each new batch or polarity change.

### Lipid identification and data preprocessing

The raw data were converted to mgf data format using proteoWizard software (22). The National Institute of Standards and Technology MS PepSearch Program was used to search the converted files against LipidBlast (23, 24) libraries in batch mode. We optimized the search parameters using the NIST11 library and LipidBlast libraries and compared them against our lipid standards. The *m/z* width was determined by the mass accuracy of internal standards and was set 0.001 for positive mode and 0.005 for negative mode. The minimum match factor used in the PepSearch Program was set to 250. The MS/MS identification results from all the files were combined using an in-house script to create a library for quantification. The class identification was verified by comparing the retention time of identified lipid to retention time of internal standard. All raw data files were searched against this library of identified lipids with mass and retention time using Multiquant 1.1.0.26 (ABsciex, Concord, Canada) (25). The oxidized lipids were also identified using Multiquant. Quantification was done using MS1 data. The missing values in the data were imputed using K-nearest neighbor (KNN) method. Internal standards were used to normalize the data to correct for the variation in instrument response because of various sources throughout an analytical assay. The normalization was performed using the crosscontribution compensating multiple internal standard normalization methods (26). The QC samples were used to remove technical outliers and lipid species that were detected below the lipid class-based lower limit of quantification. QC samples evenly distributed along analytical runs of the study were analyzed. After normalization, data from each mode was combined and the repeated lipids in each mode were removed based on their reproducibility in QC samples. In short, data are carefully manually curated after combining, and only reliable lipids and features (some marked as unknowns) are kept as final data. The average coefficient of variation of all the lipids detected in the study samples was <20%.

### Lipidomic data downstream processing

Samples were received in a single batch, with 729 lipid species detected in total. The nomenclature used for individual lipid species begins with the abbreviation of the lipid class followed by the number of carbon atoms in the molecule and then by the number of double bonds. Missing values exist widely in MS-based metabolomics data (27). Missing values affect normality and variance of data. KNN method was reported to be the best method for restoring them (28). Therefore, KNN method was used to impute missing lipid values, similar to earlier work (29). Data were then log transformed and subjected to median normalization (30), before downstream analysis.

### Source of variation analysis and data screen

The lipidomic dataset of maternal plasma has a total of 729 lipid species. In order to select features capable of distinguishing preeclampsia and control statuses, a preliminary screen was conducted based on the source of variation (SOV) analysis, to explore the contributions of different clinical/physiological factors to the overall lipidomics changes. Only lipid species with a preeclampsia/control *F* statistic value >1 were included in further analysis, which meant that for these screened lipids, the sample preeclampsia/control status had a regression sum of square larger than error sum of square. This screening process finally identified 280 such lipid species.

### Differential lipid species identification

R limma package was used to identify the differentially expressed lipids between preeclampsia/control status, with adjustment of confounders with *F* statistic >1. The lipids with *P* values <0.01 were selected into a significant list. Lipids that also associate with any confounders were removed from the list. We further used a subset of samples without gestational diabetes or smokers for differential lipid analysis similarly, in order to exclude possible confounding from smoking and gestational diabetes that only exist among the cases. Lipid species overlapped between the two significant lists were selected as the final list of differential lipid species associated with preeclampsia.

### Weighted gene coexpression network analysis

Before weighted gene coexpression network analysis (WGCNA) performance, the normalized lipid values were adjusted via limma (31). This time, the normalized value of each lipid species was predicted using preeclampsia/control status, and the confounding factors are shown in Table 1, including smoking status, baby gender, maternal ethnicity, maternal age, parity, prepregnancy BMI, gestational diabetic status, chronic hypertension status, membrane rupture status, abruption, and neonatal malformation status, and then the regression coefficient on preeclampsia/control variable plus the residual value was used to compute the adjusted lipid species value, with confounding effects regressed out. Next, the preeclampsia and control samples were separated into different groups and then analyzed by WGCNA separately (32). For both groups, the WGCNA estimated a soft threshold (power) of 4 with RsquaredCut of 0.85 and verbose of 5. Using these criteria, the WGCNA constructed modules with minModuleSize of 10, mergeCutHeight of 0.25, deepSplit of 2, and verbose of 3. Then topological overlap value between these lipid species was computed from the adjacency score as well as their connectivity values. The topological overlap value was further converted to a distance value by subtracting it from 1, providing a distance matrix covering all lipid pairs, which was next used to cluster the lipids using hierarchical clustering, and lipid modules could be identified from the resulting dendrogram. Within each module, only lipid pairs with a topological overlap value >0.5 were retained.

For the integrated analysis using all preeclampsia and control samples, the WGCNA suggests a soft threshold (power) of 8. Using the power of 8, the WGCNA constructed modules with the same of other parameters as described previously and performed module-trait association analysis.

### Classification model

To identify the best machine learning model to distinguish preeclampsia and control samples using lipidomic features, *Lilikoi* was used to construct seven different classifier models (33), including C4.5 decision tree (RPART), gradient boosting machine, random forest (RF), logistic regression with elastic net regularization, linear discriminant analysis, support vector machine, and nearest shrunken centroids (prediction analysis for microarrays). The 64 samples were first split 80/20 into training and test datasets, and then, the classifier model was trained (on the training set) using a 10-fold crossvalidation method. The best model was selected based on the *F* statistics and balanced accuracy in the test dataset. To determine if adding confounding factors would improve the classifier's performance, potential clinical confounding factors were also added in addition to the lipid features selected by the best model. In the prediction model, the *Lilikoi* parameters *dividep*, *dividseed*, and *times* were set to 0.8, 1,996, and 10, respectively.

### Lipid pathway and phenotype mapping

To map metabolic pathways to lipid species, first, their *m/z* value, ion adduct information, and lipid class information were used as a query to perform a bulk search in the LIPID MAPS database. For a query “lipid class,” all isomers with the same carbon and double bond numbers are returned as the search results. Next, the query lipid and the systematic names of these isomers were used as the input to map to standard Human Metabolome Database, PubChem, and Kyoto Encylopedia of Genes and Genomes IDs in *lilikoi.* These IDs were then used for the corresponding pathway analysis.

### Causality analysis

We sorted the 11 lipids deemed significantly associated with severe preeclampsia by time series according to the gestational ages of samples. Then, we used the lmtest package (version 0.9-37) on R platform (version 3.6.3) to perform the Granger causality test for potential causality relationships among lipids and preeclampsia. A causality interaction is significant when *P* < 0.05. Significant causality interactions were collected for further analysis.

## Results

### Overview of the study cohort and lipidomics results

**Fig. 1:**
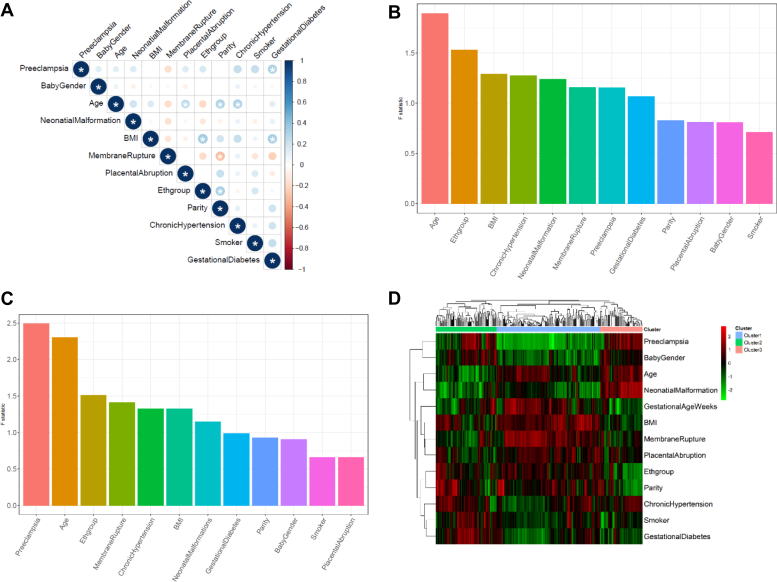
Exploratory analysis of preeclampsia and control samples. A: Correlation matrix of the phenotypic variables on the 64 samples (20 control vs. 44 preeclampsia samples). Significant correlations (*P* < 0.05) are shown with ∗. B: Source of variation (SOV) results across the 64 samples using 729 lipid species. C: SOV results across the 64 samples using 280 lipid species, which all have *F* statistic values of at least 1 (*F* statistic of the error term). D: Heat map showing the correlations between the 280 lipid species and the confounding factors. The columns are lipids, and rows are clinical factors. Each entry of the heat map represents the Pearson correlation coefficient value between the lipid and the clinical factor.

The study is a nested case and control study from a precollected population-based biobank from the University of Hawaii. Maternal plasma of 64 samples (44 severe preeclampsia patients and 20 controls) was used for this study. Table 1 shows the demographic and major clinical characteristics of the subjects. As expected, the patients with severe preeclampsia delivered significantly earlier than the controls (average gestational age of 35.82 weeks vs. 39.10 weeks, *P* = 4.80e-9). Gestational diabetes is also significantly associated with preeclampsia (*P* = 0.013), confirming previous reports that gestational diabetes is a risk factor for preeclampsia (34, 35). In addition, parity is also associated with the preeclampsia group (*P* = 0.017), as expected (36). Other risk factors, including smoking and chronic hypertension, show less than significant associations with severe preeclampsia (*P* = 0.088 and 0.085, respectively), which may be due to the limited observations of smokers and patients with chronic hypertension in this study. Beyond the correlation between preeclampsia and other clinical factors, we also performed correlation analysis among all clinical factor pairs (Fig. 1A). Supplemental results in Table 1, correlations are widely detected between preeclampsia/control status and other variables, such as gestational age (Pearson's correlation coefficient = −0.533) and gestational diabetes (Pearson's correlation coefficient = 0.307). Gestational diabetes, the other significant clinical factor sharing comobility with preeclampsia, is also correlated with other variables, such as BMI.

The untargeted lipidomic experiments were performed by Michigan Regional Comprehensive Metabolomics Resource Core, using an LC tandem MS (LC/MS/MS) lipidomics assay (see the Materials and Methods section). The resulting lipidomics dataset is composed of a total of 729 annotated lipid species. The internal controls are listed in supplemental Table S1, and the metabolomics data for both positive and negative modes are included in supplemental Table S2. The principal component analysis plot, with case/control samples, as well as pool and plasma controls is shown in supplemental Fig. S1. To identify lipids that are truly associated with preeclampsia (rather than because of other confounders), a preliminary screen was conducted on the lipid species using the SOV analysis. A total of 280 lipid species with preeclampsia/control *F* statistic values >1 were selected for subsequent analyses. As a confirmation, the ranking of preeclampsia/control status is improved from the seventh in the whole dataset (Fig. 1B) to the first in the filtered metabolomics subset with 280 lipids (Fig. 1C).

We next examined the correlations between the clinical variables and the 280 screened lipid species by heat map (Fig. 1D). Hierarchical clustering analysis on the 280 lipid species shows three main clusters. Clusters 1, 2, and 3 are composed of 179, 52, and 49 lipid species, respectively. Cluster 2 is significantly enriched in diacylglycerol lipids (*P* = 7.90e-3; odds ratio = 3.02) and PE (*P* = 4.38e-2; odds ratio = 2.39). Cluster 3 has a large enrichment in two kinds of OxPLs: oxidized phosphatidylethanolamine (OxPE; *P* = 9.35e-6, odds ratio = 5.71) and oxidized PC (OxPC, *P* = 3.04e-4, odds ratio = 5.71) but have a significant reduction in the level of TAG (*P* = 1.66e-2, odds ratio = 0.14). No lipid species is detected as significantly enriched in cluster 1. In summary, the results provide the initial evidence that lipidomic changes are associated with severe preeclampsia, despite the complexity because of other clinical variables. The complex correlations between preeclampsia and other clinical variables suggest that their relationships may be mediated through molecules including lipids studied here.

### Correlation network analysis of lipidomics in relation to preeclampsia

**Fig. 2:**
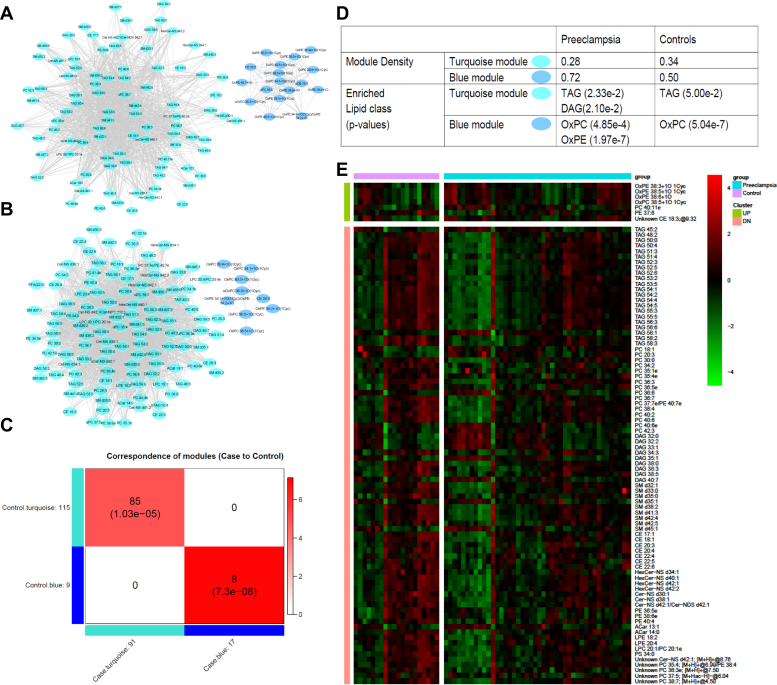
WGCNA network comparison between preeclampsia and control samples. A and B: WGCNA network in preeclampsia (A) and control (B), respectively. Each node represents a lipid species. C: Overlap between modules of networks in control and preeclampsia samples. D: Table showing more properties of modules in networks of control versus preeclampsia samples, including module density and enriched lipids in each module. E: Heat map of lipids with significant connectivity difference in WGCNA networks (A and B), between control versus preeclampsia. Connectivity value is defined as increased or decreased if the connectivity value in the preeclampsia network minus that in the control network is larger than 5 or less than −5, respectively.

To elucidate the relationships between lipidomics and severe preeclampsia, we next constructed the correlation networks for preeclampsia and control samples separately, using WGCNA method on the 280 screened lipid species (32). As shown in Fig. 2A–D, in both preeclampsia and control conditions, the networks contain TAG-enriched module (turquoise color) and OxPL-enriched module (blue color). The TAG-enriched modules in both the preeclampsia and control group correspond well with significant overlap, so does the OxPL-enriched module (Fig. 2C). However, the OxPL-enriched network module is more densely connected in preeclampsia (density = 0.72) than in control (density = 0.50). Moreover, the enrichment of OxPE only shows in preeclampsia but not in the control samples, as shown in Fig. 2D. Confirming this, a combined WGCNA analysis using both preeclampsia and control samples shows significant positive association (*P* = 0.03, correlation = 0.28) of OxPL-enriched module with preeclampsia (supplemental Fig. S2). OxPLs have been previously associated with a variety of diseases, including arteriosclerosis, diabetes, and cancers (37). The increase in both the enrichment of OxPLs as well as their interconnections suggest that preeclampsia is another OxPL-related disorder. On the other hand, the TAG-enriched turquoise network module has lower connection density in preeclampsia (density = 0.28) than in control (density = 0.34).

To further explore the lipid correlation differences between preeclampsia and control samples, we specifically extracted lipid species with significant changes in network connectivity values. We obtained the network connectivity value for every node, by summing over the weights (weighted correlations) of all edges from a node. Then, we identified lipids with connectivity changes between case and control conditions of greater than 5 (lipids with increased connectivity) or less than −5 (lipids with decreased connectivity). With such a stringent cutoff threshold, seven lipids are identified with increased connectivity, and 84 lipids are with decreased connectivity (Fig. 2E). TAG and diacylglycerol are the only two enriched lipid classes with decreased connectivity (Fig. 2D, E), suggesting that their biogenesis processes are disrupted in preeclampsia. Of the seven lipids with increased connectivity in preeclampsia, four are OxPE or OxPC lipids, corroborating the earlier WGCNA results of increased module density and lipid enrichment (Fig. 2A–C). Together, these results show that oxidative lipid genesis is enhanced in severe preeclampsia.

### Lipids and their pathways associated with severe preeclampsia

**Fig. 3:**
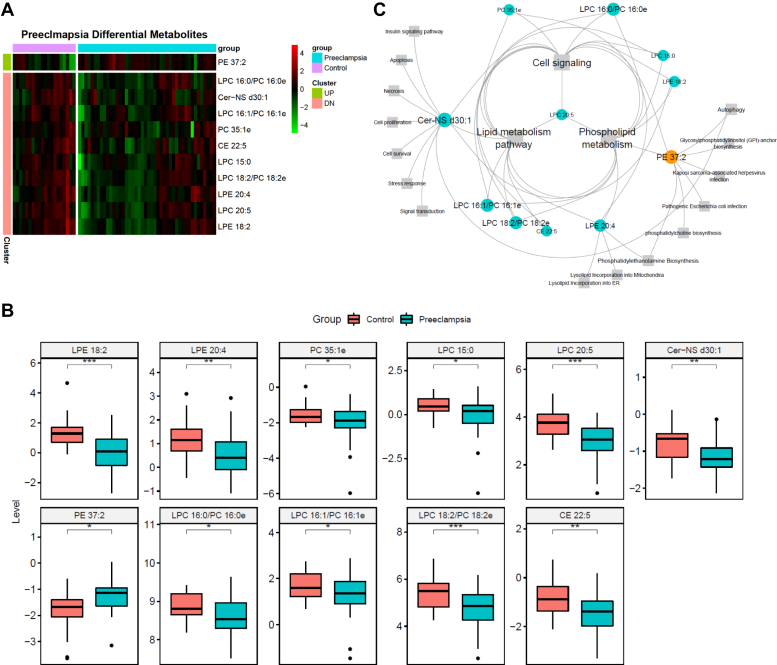
Lipids show significantly different levels in preeclampsia and control samples. A: Heat map of the 11 lipids with significant difference between preeclampsia and control samples. B: Box plots of the 11 lipids in our lipidomics data. C: Bipartite graph of lipids in (A) and their affiliated metabolic pathways. Elliptical nodes: lipid. Rectangular nodes: pathways from HMDB, PubChem, and KEGG databases. Blue color: downregulation in preeclampsia. Orange color: upregulation in preeclampsia. Note: lipids without any metabolomic pathway affiliations are omitted. The unseparated LPCs/PCs are shown in the same plot. ∗*P* < 0.05, ∗∗*P* < 0.01, ∗∗∗*P* < 0.001.

To exclude effects from potential confounding factors, we also analyzed the overall lipid concentration changes by adjusting for confounding. Since the SOV analysis shows six potential confounders (Fig. 1C), we conducted generalized linear regression using severe preeclampsia condition and six other clinical confounders. As a result, 28 lipid species are identified as significantly (*P* < 0.01) different regarding the severe preeclampsia condition (supplemental Fig. S3). Among them, nine lipids also have significant associations with other confounders (*P* < 0.01), leaving 19 lipids significantly different because of severe preeclampsia condition only. Since smoking (n = 7) and gestational diabetes (n = 11) only appear in cases (Table 1), we also used the subset of 46 samples that are nonsmokers without gestational diabetes for differential lipid analysis, with adjustment for confounding (supplemental Fig. S4). The subset-based (n = 46) analysis yielded 28 lipids significantly (*P* < 0.01) different in the severe preeclampsia condition (supplemental Fig. S5). Among them, five lipids also have significant (*P* < 0.01) association with gestational age and ethnicity, leaving 23 lipids uniquely associated with severe preeclampsia. Finally, we intersected the unique 19 lipids in the full set and the unique 23 lipids in the subset of 46 samples and obtained 11 lipids that are highly and specifically associated with severe preeclampsia (Fig. 3A). There are two LPCs: LPC 15:0 and LPC 20:5; PC 35:1e; two lysophosphatidylethanolamines (LPEs): LPE 18:2 and LPE 20:4; CE 22:5, Cer (Cer-NS) d30:1, LPE (PE) 37:2; and three LPCs/PCs: LPC 16:0/PC 16:0e, LPC 16:1/PC 16:1e, LPC 18:2e/PC18:2e. The LPCs/PCs are the lipids with similar spectrum that their existence can be identified by the MS2 spectrum, but their quantity cannot be separated by the MS1 spectrum, which is used for lipid quantification. Most of the 11 lipids are downregulated in severe preeclampsia, except PE 37:2 (Fig. 3A, B and supplemental Table S3). Among the 10 downregulated lipids, LPE 18:2, LPE 20:4, CE 22:5, Cer-NS d30:1, and PC35:1e also have reduced network connectivities in WGCNA, suggesting their dual attenuations on both lipid concentrations and correlations.

To understand the functional role of the lipids with significantly different concentrations only because of preeclampsia versus control, we attempted pathway enrichment analysis on the 11 lipids. However, this task was not easy, as the current shotgun lipidomic technique can only identify lipids by their class group and total number of carbons and double bonds, rather than providing definitive and unique identifications. To overcome this issue, we performed isomer searches of each lipid species first, then used all the lipid isomers together to search for the corresponding Human Metabolome Database, PubChem, and Kyoto Encylopedia of Genes and Genomes pathways (see the Materials and Methods section). As a result, all 11 lipid species yielded associated pathways (Fig. 3C). Cer Cer-NS d30:1 is overall reduced in preeclampsia samples. Cer is involved in various signaling pathways, including insulin signaling pathway, cell apoptosis, and stress response (Fig. 3C). PCs and LPCs are overall reduced in preeclampsia, and they are linked to phospholipid (PL) metabolism. Decreased PL metabolism is associated with preterm delivery, a major clinical feature in preeclampsia (38). On the other hand, as the only lipid increased in preeclampsia, PE 37:2 is linked to various infection and immune response pathways, including “pathogenic *Escherichia coli* infection” and autophagy process, both of which increase risk of preeclampsia (39, 40).

### A metabolomics-based preeclampsia biomarker model

**Fig. 4:**
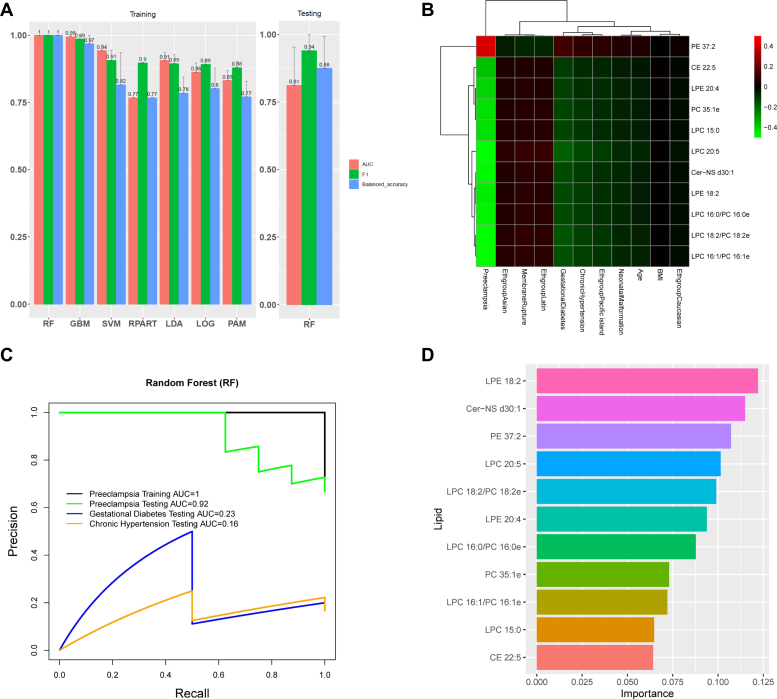
Biomarker classification model for preeclampsia. A: Performance of machine learning models on predicting severe preeclampsia using 11 potential lipid markers. Comparison of seven popular classification models on training data (left). From left to right: random forest (RF), gradient boosting (GBM), support vector machine (SVM), linear discriminant analysis (LDA), elastic net (LOG), decision tree (RPART), and nearest shrunken centroids (PAM). The performance on testing data from the winning method RF, based on training data, is shown on the right. All samples were randomly split into training data (80%) and testing data (20%) 10 times. The average value and standard error are shown for three performance metrics: area under the ROC curve (AUC), F1 statistic, and balanced accuracy. B: Heat map of correlation coefficients between 11 potential lipid markers and clinical variables. C: Precision-recall curve of RF model using 11 markers on: training data for severe preeclampsia and testing data for severe preeclampsia, gestational diabetes, and chronic hypertension, respectively. D: Normalized variable importance scores for the 11 lipid markers in the RF model.

An important application of lipidomics is to screen for potential disease biomarkers. To achieve this, we split samples with 80/20 ratio as training and test datasets, respectively. We performed feature selection among the 11 lipids, using a criteria of information gain value greater than 0.1. We compared the performance of several popular machine learning algorithms in *Lilikoi* R package in the test dataset to obtain the best classification methods for the lipidomics data (Fig. 4A). These classification algorithms include linear discriminant analysis, RFs, logistic regression with elastic net regularization, gradient boosting machine, support vector machine, nearest shrunken centroids (prediction analysis for microarrays), and decision tree (RPART). We used F1 statistics and balanced accuracy metrics to evaluate the models, given the unbalanced size of the preeclampsia and control samples. RF is selected as the final best model from the training dataset, and it yields the F1 statistic, area under the receiver operating characteristic curve and balanced accuracy of 0.94, 0.81, and 0.88, respectively, in the test dataset (Fig. 4A). The 11 lipids in the RF model show high correlations with preeclampsia but not any other clinical confounders (Fig. 4B), suggesting that they are biomarkers specific to preeclampsia. To confirm this, we used this classification model built for preeclampsia to predict its classification capability for other confounders, using the testing dataset. For gestational diabetes and chronic hypertension classification, it yields area under the receiver operating characteristic curve of 0.23 and 0.16, respectively, in the precision-recall curves (Fig. 4C), confirming the specificity of the 11 lipid-biomarker model for severe preeclampsia. Among the 11 lipid biomarkers, 10 belong to cluster 1 in Fig. 1D, where the lipids are predominantly negatively correlated with preeclampsia, and PE 37:2 is the only lipid belonging to cluster 2 in Fig. 1D. According to the feature importance ranking, LPE 18:2, Cer-NS d30:1, and PE 37:2 are the top three most important lipids predictive of severe preeclampsia, with the scaled feature important scores of 0.122, 0.115, and 0.107.

### Predicted causality relationships among lipids and preeclampsia

**Fig. 5:**
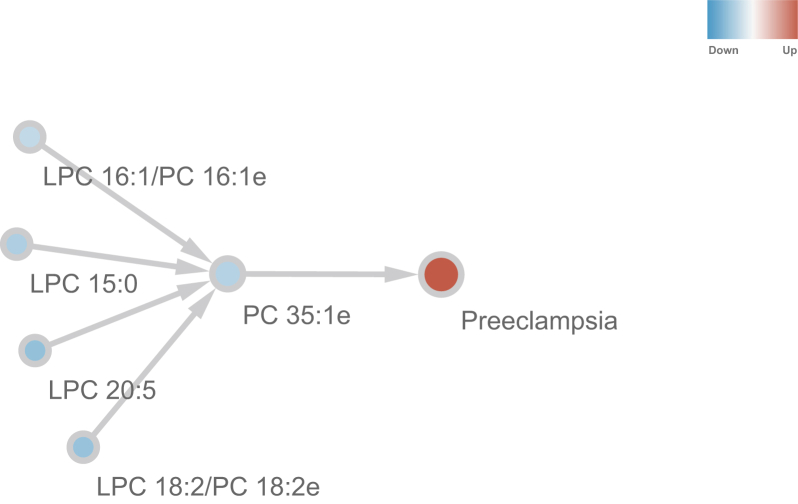
Predicted causality interactions among lipids and preeclampsia. Edges are from causes to results. Blue nodes are downregulated lipids, whereas red one is preeclampsia. Only significant (*P* < 0.05) causality interactions are shown. No significant causality interaction was found for upregulated lipid.

Beyond the aforementioned correlation analysis, we also explored the potential causal relationship between the 11 lipids and severe preeclampsia, using a Granger causality test (41). The results show significant (*P* < 0.05) causality interactions from LPC 16:1/PC 16:1e, LPC 18:2/PC 18:2e, LPC 15:0, LPC 16:1, LPC 18:2, LPC 20:5 to PC 35:1e, and then from PC 35:1e to severe preeclampsia (Fig. 5). It is interesting to see causal interactions from PC of smaller molecular weights (PC 16:1e and PC 18:2e) to that of larger molecular weights (PC 35:1e). Since LPC can be converted to and from PC, there appears the “pulling effect” from substrate LPCs (LPC 15:0, LPC 16:1, LPC 18:2, and LPC 20:5) to PC (PC 35:1e).

## Discussion

Preeclampsia is a complex and heterogeneous disorder of pregnancy (1). To improve our understanding of severe preeclampsia, we conducted a lipidomics study on a unique multiethnic cohort in Hawaii. To exclude the potential impact of mixed ethnicity as well as other confounding factors, we excluded lipids that are associated with any of these confounding factors in the linear regression model. As a result, among 30 significantly differential lipid species associated with severe preeclampsia, 11 lipids have exclusive associations with severe preeclampsia but not any other clinical features.

One of the most significant and novel findings in this study is the increased correlation and intensities among OxPE and OxPC lipids in preeclampsia. Similar increases of OxPLs have been observed and associated with various other diseases, such as arteriosclerosis, diabetes, and cancer (37). OxPLs have a known close relationship with oxidative stress. In preeclampsia, hypoxia is one of the most important features and a source of oxidative stress (42). This hypoxic condition in preeclampsia is derived from the incomplete remodeling of spiral arteries by extracellular trophoblasts in this disease. Furthermore, such spiral arteries cannot provide adequate blood to the placenta, resulting in a hypoxic condition (43). Thus, we speculate that enhancement of OxPL correlation is the result of oxidative stress induced by hypoxia.

**Fig. 6:**
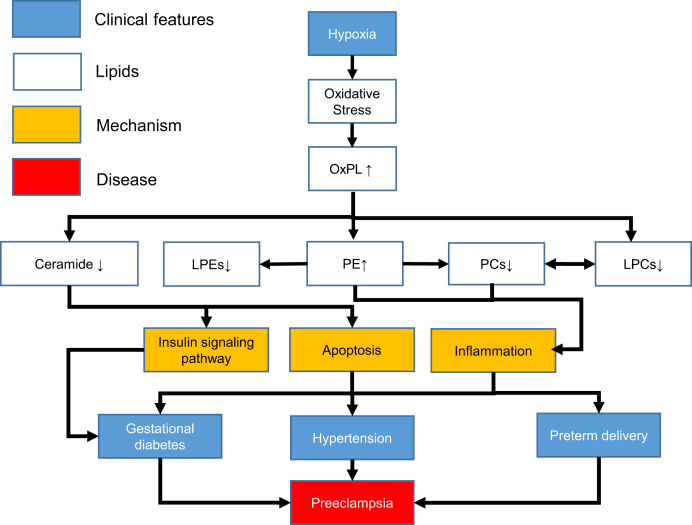
A proposed model of lipidomic changes in the pathogenesis of severe preeclampsia.

Changes in several lipid classes may contribute to multiple clinical features of severe preeclampsia (Fig. 6). The first link is from the increase of PE 37:2, which mediates the pathways of pathogenic *E. coli* infection as well as autophagy processes (Fig. 3C), both of which have a close relationship to inflammation (39) and have been implicated in the mechanisms responsible for gestational diabetes (44), hypertension (45, 46), and preterm delivery (47, 48). This lipid can specifically interact with the bundle-forming pilus of pathogenic *E. coli* for bacterial autoaggregation and adherence to host cells and contribute to infection (49). In autophagy process, the microtubule-associated protein 1A/1B-light chain 3 (LC3) in cytosol can conjugate to PE to form LC3-PE conjugate (LC3-II), which is recruited to autophagosomal membranes (50). LC3-II was shown to increase in placenta, indicating an increased autophagic activity during the pathogenesis of this disorder (51). Second, Cer (Cer-NS d30:1) is also decreased in severe preeclampsia (Fig. 3A). Cer is a sphingolipid bioactive molecule that induces apoptosis and other forms of cell death and triggers autophagy (52) (Fig. 3C). Cer is also involved in the mechanism of diabetes by regulating the insulin signaling pathway as a second messenger (53). Alteration of Cer is associated with hypertension (54), presumably through apoptosis/cell death. Third, LPCs and PCs are significantly reduced in severe preeclampsia (Fig. 3A). PCs are the main PLs of cell membranes (up to 50%), and their downregulation likely causes cell membrane damage, which can contribute to preeclampsia by maternal/fetal tissue injuries and increasing the risks of infection and inflammation (55). LPEs, LPCs, and PCs are involved in PL metabolisms. Interestingly, reduced PL metabolisms are significantly associated with preterm delivery, a major clinical feature of severe preeclampsia (38).

Comparisons among our study with previous lipidomics preeclampsia studies reveal certain degree of consistency, such as lower levels in neutral lipids such as sterols (17), reduction in Cer (19), increased oxidized lipids (e.g., OxPC) (18). One earlier lipidomics biomarker study was particularly interesting (18), where serums of mostly Caucasian women were collected in 12–14 weeks of pregnancies. The authors had a discovery set of 27 controls and 29 preeclampsia patients and a validation set of 43 controls and 37 preeclampsia cases. They identified 23 potential biomarkers for early onset preeclampsia, among them the largest lipid class was PC. Although not the same lipids, we identified four PCs as potential biomarkers for severe preeclampsia. One major advantage of our study compared with this study is the abundance of annotated lipids, which helps to reconstruct the lipidomic landscape bioinformatically, allowing much better understanding of the metabolic mechanisms beyond biomarkers. In addition, the difference of lipid biomarkers between two studies can also be explained by different objectives: severe preeclampsia versus early onset preeclampsia. Another study on placental lipid profiles from 23 preeclampsia pregnancies showed higher neutral lipid content than 68 healthy controls (40% higher TAG and 33% higher CE) as well as increases in most PC lipid species. The authors concluded that placenta has a lipid storage status under preeclampsia condition (56). Their result in placentas is almost completely complementary to our maternal blood observations (57), suggesting a “source and sink” scenario at play, that is, the deprivation of these lipids in the blood supplies the lipid storage in preeclamptic placentas.

In summary, our study highlights the lipidomic changes manifested in severe preeclampsia patients and points to plausible lipid metabolic mechanisms. We have identified a close relationship between OxPLs and preeclampsia, presumably through the oxidative stress mechanisms because of hypoxia. We propose that the decreases in many lipids (e.g., LPCs, PCs, and LPEs) in serum are potential specific markers for severe preeclampsia, and their changes can be explained by the PL-centered lipidomic axis. These molecular changes coherently mediate dysregulation in biological functions, such as insulin signaling, immune response and PL metabolism.

## Data availability

Raw data, including internal controls, are listed in supplemental Table S2. All scripts to analyze the metabolomics data are available at github: https://github.com/lanagarmire/preeclampsa_lipidomics.

## Supplemental data

This article contains supplemental data.

## Conflict of interest

The authors declare that they have no conflicts of interest with the contents of this article.
