# Supplementary material for: The maternal blood lipidome is indicative of the pathogenesis of severe preeclampsia
Source: J Lipid Res. 2021 Sep 20;62:100118. doi: 10.1016/j.jlr.2021.100118 (PMC8503628; doi:10.1016/j.jlr.2021.100118)
Supplement: Supplemental Table S1 [file mmc1.docx]

**Supplementary Table 1.** The spiking internal standards used in this study.

| **Internal Standards** | **PubChem CID** | **Molecular Formula** | **Molecular Weight** |
| --- | --- | --- | --- |
| 1-heptadecanoyl-2-hydroxy-*sn*-glycero-3-phosphocholine LPC (17:0/0:0) | 24779463 | C25H52NO7P | 509.7 |
| 1,2-diheptadecanoyl-sn-glycero-3-phosphocholine PC (17:0/17:0) | 24778784 | C42H84NO8P | 762.1 |
| 1,2-diheptadecanoyl-sn-glycero-3-phosphoethanolamine PE (17:0/17:0) | 46891875 | C39H78NO8P | 720.0 |
| 1,2-diheptadecanoyl-*sn*-glycero-3-phospho-L-serine (sodium salt) PS (17:0/17:0) | 46891782 | C40H77NNaO10P | 786.0 |
| N-heptadecanoyl-D-*erythro*- sphingosylphosphorylcholine 17:0 SM (d18:1/17:0) | 46891763 | C40H81N2O6P | 717.1 |
| cholest-5-en-3ß-yl heptadecanoate 17:0 cholesteryl ester | 242083828 | C44H78O2 | 639.1 |
| 1-palmitoyl-2-oleoyl-*sn*-glycerol 16:0-18:1 DG | 5282283 | C37H70O5 | 594.9 |
| 1-heptadecanoyl-rac-glycerol 17:0 MG | 107036 | C20H40O4 | 344.5 |
| 1,2,3-triheptadecanoyl-glycerol Triheptadecanoate 17:0 TAG | 3625612 | C54H104O6 | 849.4 |
| N-heptadecanoyl-D-*erythro*-sphingosine C17 Ceramide (d18:1/17:0) | 136212696 | C41H82N2O11S | 811.2 |
| 1,2-diheptadecanoyl-*sn*-glycero-3-phosphate (sodium salt) 17:0 PA | 99344107 | C37H72O8PNa | 698.9 |
| 1,2-diheptadecanoyl-*sn*-glycero-3-phospho-(1'-*rac*-glycerol) (sodium salt) 17:0 PG | 99344100 | C40H78O10PNa | 773.0 |
| 1-heptadecanoyl-2-(5Z,8Z,11Z,14Z-eicosatetraenoyl)-sn-glycero-3-phospho-(1'-myo-inositol) (ammonium salt) 17:0-20:4 PI | 9547140 | C46H84NO13P | 890.1 |
| 1,3(d5)-dinonadecanoyl-2-hydroxy-glycerol DG d^5^-(19:0/0:0/19:0) | 9543978 | C41H80O5 | 658.1 |
| Glyceryl tri(palmitate-d^31^)  TG d^31^ | 329760162 | C51D93H5O6 | 900.9 |
