# Supplementary material for: The maternal blood lipidome is indicative of the pathogenesis of severe preeclampsia
Source: J Lipid Res. 2021 Sep 20;62:100118. doi: 10.1016/j.jlr.2021.100118 (PMC8503628; doi:10.1016/j.jlr.2021.100118)
Supplement: Supplemental Table S3 [file mmc3.docx]

**Supplementary Table 2**: Summary of 28 lipids with significant changes in severe preeclampsia.

| **Lipid class** | **Lipid species** | **logFC** | **P.Value** | **Marker*** |
| --- | --- | --- | --- | --- |
| cholesterol ester | CE 20:3 | -0.53629 | 0.00342 | No |
| cholesterol ester | CE 22:4 | -0.92434 | 0.0016 | No |
| cholesterol ester | CE 22:5 | -0.82596 | 0.001532 | Yes |
| ceramide | Cer-EODS d55:2 | -0.68217 | 0.007948 | No |
| ceramide | Cer-EOS d49:1 | -0.54243 | 0.007867 | No |
| ceramide | Cer-NDS d36:0 | -0.4548 | 0.004537 | No |
| ceramide | Cer-NS d30:1 | -0.42519 | 0.004794 | Yes |
| ceramide | Cer-NS d32:1 | -0.52777 | 0.002567 | No |
| ceramide | Cer-NS d36:1 | -0.43346 | 0.005535 | No |
| ceramide | Cer-NS d36:2 | -0.53271 | 0.006476 | No |
| ceramide | Cer-NS d38:1 | -0.75478 | 0.005235 | No |
| ceramide | Cer-NS d38:2 | -0.40489 | 0.007351 | No |
| lysophosphatidylcholine | LPC 15:0 | -0.85067 | 0.006623 | Yes |
| lysophosphatidylcholine/phosphatidylcholine | LPC 16:0/PC 16:0e | -0.41722 | 0.005 | Yes |
| lysophosphatidylcholine/phosphatidylcholine | LPC 16:1/PC 16:1e | -0.75098 | 0.005044 | Yes |
| lysophosphatidylcholine/phosphatidylcholine | LPC 18:2/PC 18:2e | -0.85908 | 0.000876 | Yes |
| lysophosphatidylcholine | LPC 20:3 | -0.49619 | 0.008334 | No |
| lysophosphatidylcholine | LPC 20:5 | -0.92406 | 0.00029 | Yes |
| lysophosphatidylethanolamine | LPE 18:2 | -1.39453 | 0.000334 | Yes |
| lysophosphatidylethanolamine | LPE 20:3 | -0.86142 | 0.000784 | No |
| lysophosphatidylethanolamine | LPE 20:4 | -0.89239 | 0.002124 | Yes |
| phosphatidylcholine | PC 20:5e | -1.80881 | 0.009567 | No |
| phosphatidylcholine | PC 35:1e | -0.77395 | 0.006537 | Yes |
| phosphatidylcholine | PC 36:3e | -0.46279 | 0.0041 | No |
| phosphatidylethanolamine | PE 37:2 | 0.580443 | 0.009559 | No |
| sphingomyelin | SM d36:0 | -0.53654 | 0.008044 | No |
| sphingomyelin | SM d36:1 | -0.47988 | 0.001424 | No |
| sphingomyelin | SM d40:5 | -0.55507 | 0.009323 | No |

* Markers for pure severe preeclampsia.
