# Supplementary material for: The maternal blood lipidome is indicative of the pathogenesis of severe preeclampsia
Source: J Lipid Res. 2021 Sep 20;62:100118. doi: 10.1016/j.jlr.2021.100118 (PMC8503628; doi:10.1016/j.jlr.2021.100118)
Supplement: Supplemental Figures S1–S5 [file mmc4.docx]

**Supplementary Figures**

**Supplementary Fig. S1.** 3D PCA plot of normal control and case samples, as well as test pool controls and pooled plasma controls.

**Supplementary Fig. S2.** WGCNA network in all samples. (A)WGCNA network of lipids data using both control and preeclampsia together. Each node represents a lipid species. Nodes in the same modules are labeled with the same color. (B) Module-trait/disease relationships.

**Supplementary Fig. S3.** Heatmap of 28 lipids in the full data set that are significantly different due to severe preeclampsia. The 19 lipids that are significantly different due to severe preeclampsia only are marked with *.

**Supplementary Fig. S4.** Source of variation (SOV) results in the subset of 46 samples that are non-smokers without gestational diabetes.

**Supplementary Fig. S5.** Heatmap of 28 lipids associated with severe preeclampsia condition in the subset of 46 samples that are non-smokers without gestational diabetes. The 23 lipids that are uniquely associated with severe preeclampsia are marked with *.


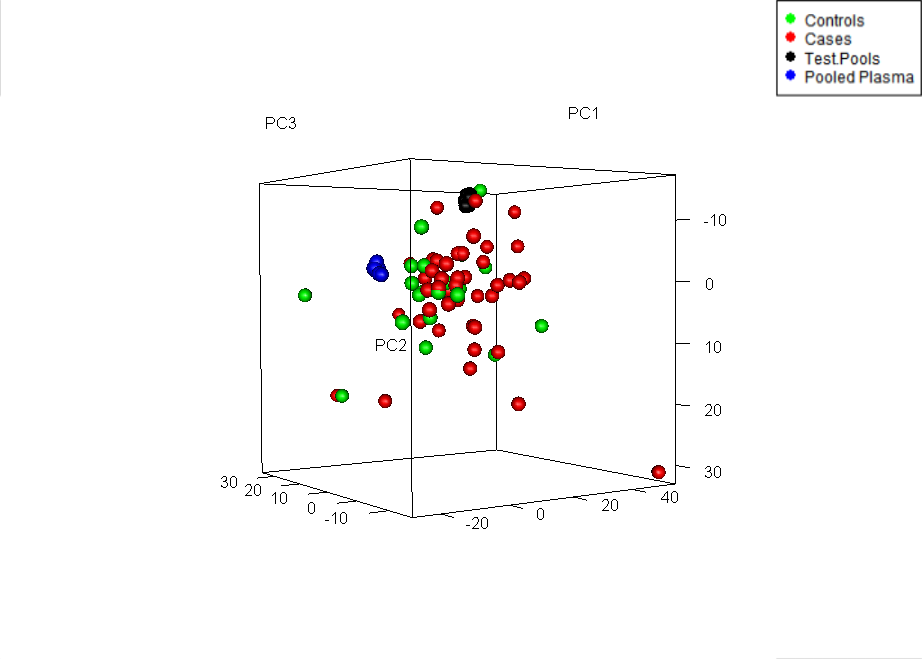


**Supplementary Fig. S1.** 3D PCA plot of normal control and case samples, as well as test pool controls and pooled plasma controls.


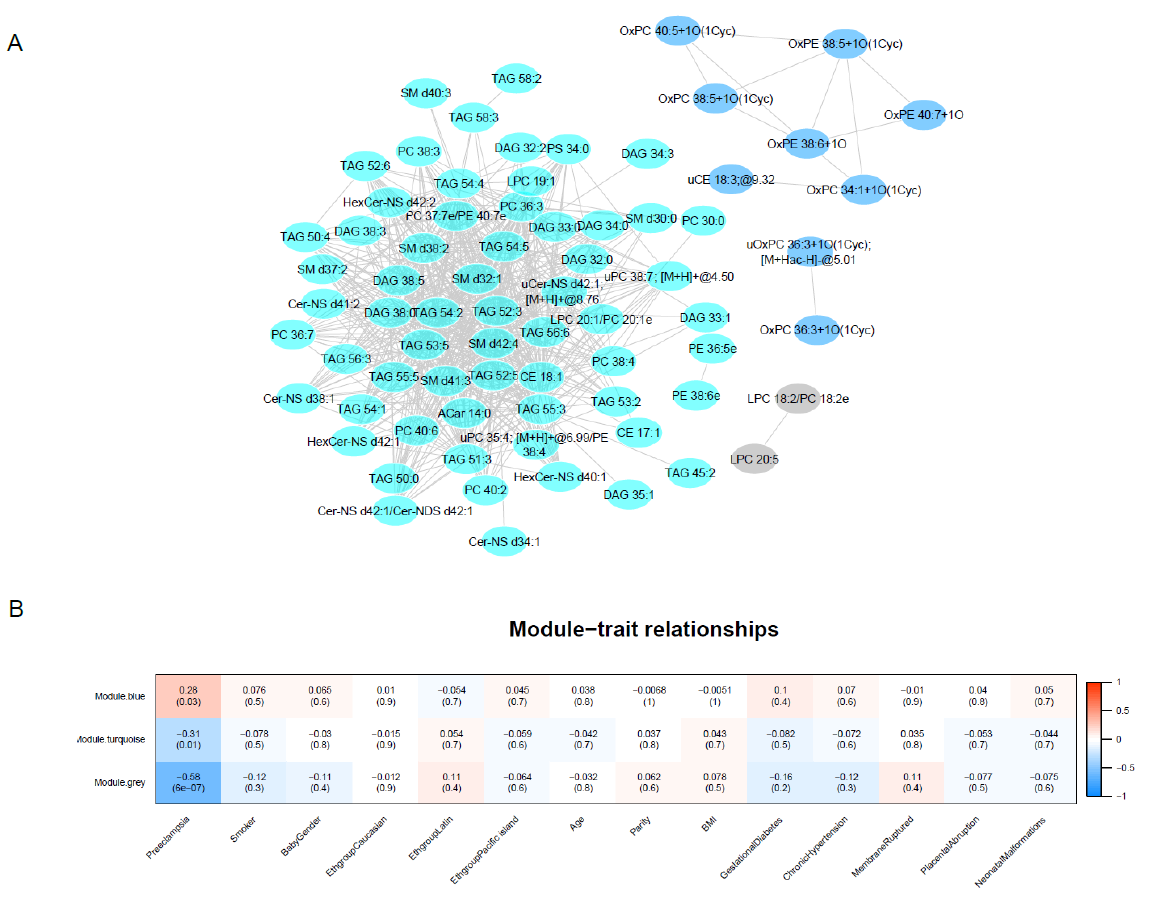


**Supplementary Fig. S2.** WGCNA network in all samples. (A)WGCNA network of lipids data using both control and preeclampsia together. Each node represents a lipid species. Nodes in the same modules are labeled with the same color. (B) Module-trait/disease relationships.


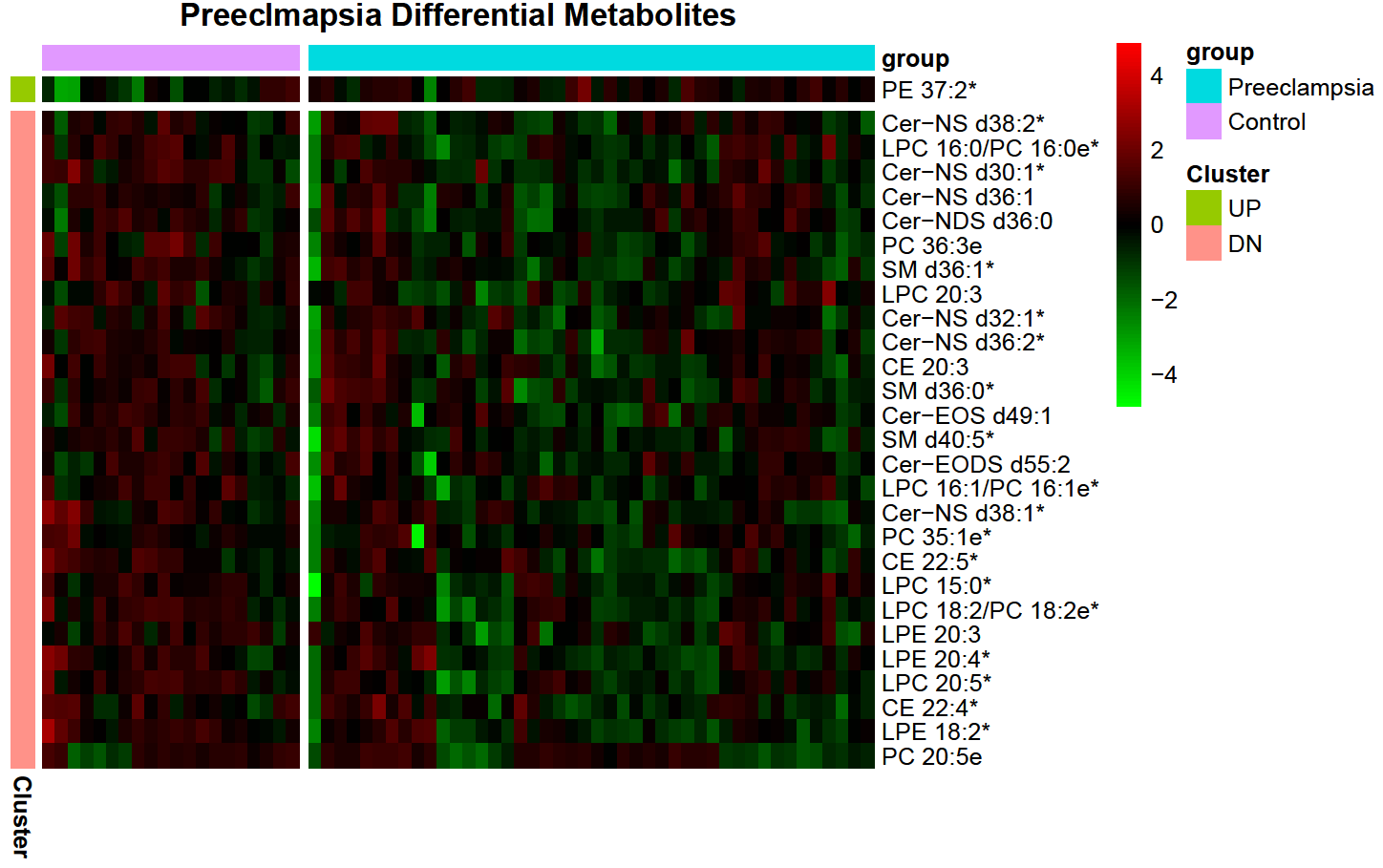


**Supplementary Fig. S3.** Heatmap of 28 lipids in the full data set that are significantly different due to severe preeclampsia. The 19 lipids that are significantly different due to severe preeclampsia only are marked with *.


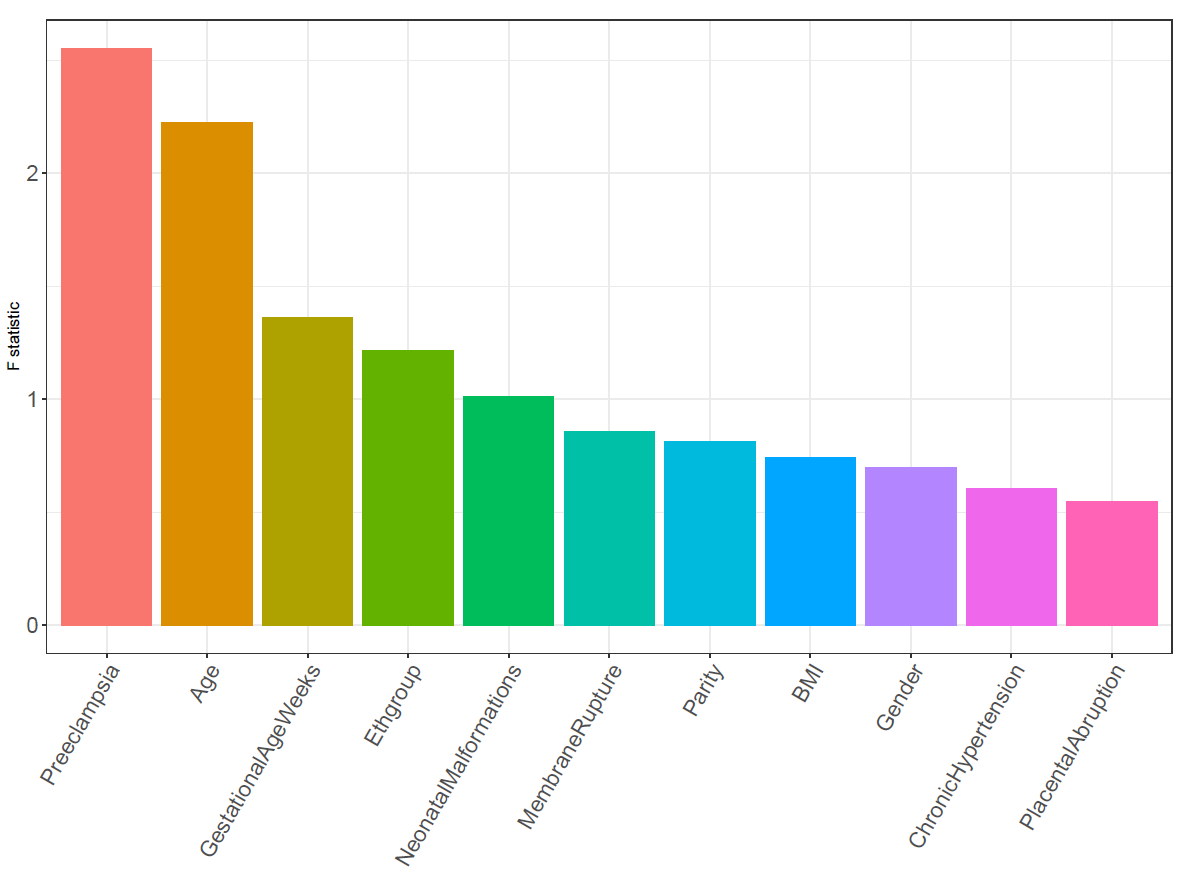


**Supplementary Fig. S4.** Source of variation (SOV) results in the subset of 46 samples that are non-smokers without gestational diabetes.


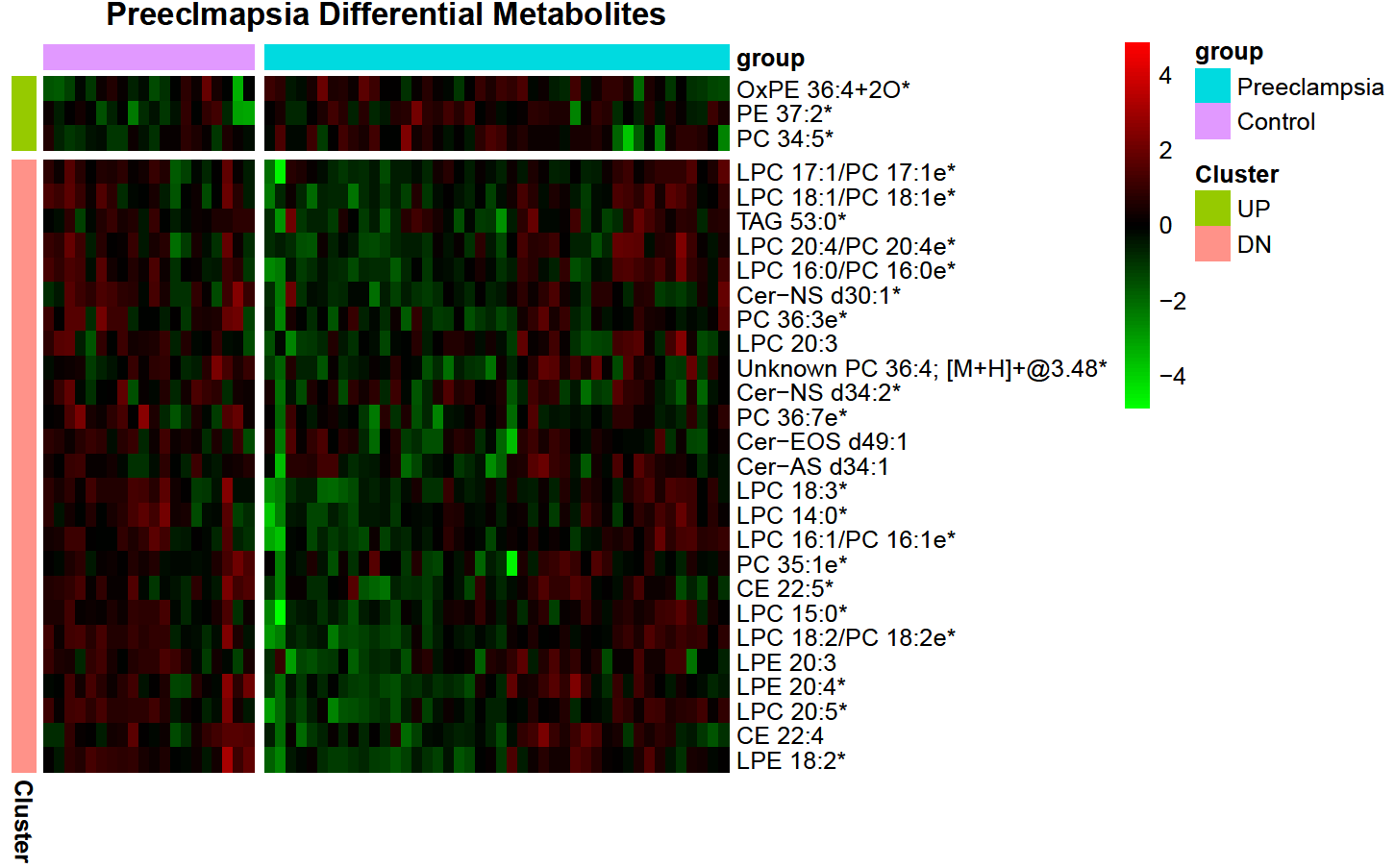


**Supplementary Fig. S5.** Heatmap of 28 lipids associated with severe preeclampsia condition in the subset of 46 samples that are non-smokers without gestational diabetes. The 23 lipids that are uniquely associated with severe preeclampsia are marked with *.
